# Associations Between Chronotype and Pain, Sleep Quality, Depression, and Quality of Life in Patients with Tension-Type Headache

**DOI:** 10.3390/healthcare13222902

**Published:** 2025-11-14

**Authors:** Emine Kılıçparlar Cengiz, Yasemin Ekmekyapar Fırat, Barış Yılbaş, Süleyman Dönmezler

**Affiliations:** 1 Department of Neurology, School of Medicine, Gaziantep Islam Science and Technology University, Gaziantep 27000, Türkiye; 2 Department of Neurology, School of Medicine, Sanko University, Gaziantep 27000, Türkiye; yaseminekmekyapar@gmail.com; 3 Department of Psychiatry, School of Medicine, Sanko University, Gaziantep 27000, Türkiye; barisyilbas@gmail.com (B.Y.); suleymandonmezler@icloud.com (S.D.)

**Keywords:** tension-type headache, chronotype, pain intensity, depressive symptoms, sleep quality, quality of life

## Abstract

Background: Chronotype refers to an individual’s preferred timing of activity and rest within a 24-h period, reflecting behavioral manifestations of the endogenous circadian rhythm. Variations in circadian timing may contribute to the temporal characteristics and pathophysiology of tension-type headache (TTH). TTH is the most common primary headache disorder and can have a significant impact on quality of life. While chronotype has been shown to influence pain perception, mood, and sleep quality in various chronic pain conditions, its relationship to TTH remains insufficiently explored. Aim: We aimed to determine the distribution of chronotypes among patients with TTH and to assess their associations with pain characteristics, depression, sleep quality, and quality of life. Methods: This cross-sectional study involved 77 adult patients diagnosed with TTH according to the International Classification of Headache Disorders (ICHD)-III criteria. Patients were recruited from the neurology outpatient clinic at SANKO University Hospital between June 2021 and June 2022. Data were collected using the Morningness–Eveningness Questionnaire (MEQ), the Visual Analogue Scale (VAS), the Hospital Anxiety and Depression Scale (HADS), the Pittsburgh Sleep Quality Index (PSQI), and the Short Form-36 (SF-36). Chronotypes were categorized as morning, intermediate, or evening. Group differences were analyzed using ANOVA, Kruskal–Wallis and linear regression models. Results: The mean age of the study sample was 29.0 [24.0–35.0] years. Fifty-five participants (71.4%) had an intermediate chronotype, 14 (18.2%) had a morning chronotype, and 8 (10.4%) had an evening chronotype. Those with an evening chronotype had a significantly lower BMI than those with an intermediate chronotype (*p* = 0.035) and lower scores on the SF-36 Role Limitations due to Physical Problems domain than those with a morning chronotype (*p* = 0.039). Chronotype (as assessed by the MEQ) was negatively correlated with sleep quality, with evening chronotypes showing poorer PSQI scores. No significant differences were found in VAS (pain intensity) and HADS (depression) scores among chronotypes. Linear regression analyses indicated that chronotype significantly predicted SF-36 Bodily Pain scores, whereas sex significantly predicted VAS pain intensity (*p* = 0.001). Conclusions: Evening chronotype is associated with poorer sleep quality and greater role limitations due to physical problemsin patients with TTH, which can potentially exacerbate the disabilities associated with headaches. Tailored interventions targeting chronotype and sleep may improve quality of life in this population.

## 1. Introduction

Tension-type headache (TTH) represents the most common form of primary headache, affecting individuals across all age groups and frequently impairing daily functioning and work productivity [1]. For TTH, studies on frequency and duration of attacks showed that affected people spend, on average, 4.7% of their time with headache [2]. Despite its high prevalence, TTH remains under-recognized as a significant public health problem. Moreover, mounting evidence indicates that sleep disturbances may lower pain threshold and amplify pain sensitivity [3]. However, there is limited research examining the relationship between chronotype and TTH.

Chronotype is generally defined as an individual’s preferred timing of activity and rest over a 24-h period. It reflects the endogenous circadian rhythm and how it synchronizes (entrains) to the 24-h cycle [4,5]. Chronotype is influenced by multiple factors, including sex, age, genetic, environmental, and psychosocial components [6]. Chronobiological research has shown that individuals can be classified as morning, evening, or intermediate chronotypes based on their sleep–wake cycles, body temperature rhythms, and hormonal secretion patterns (e.g., cortisol and melatonin) [7]. Recent studies have suggested that evening chronotype may be associated with more pronounced depressive symptoms, poorer sleep quality, and even increased pain severity in chronic pain conditions such as migraine and fibromyalgia [8,9]. The evening type is often considered more vulnerable, as it has been associated with psychiatric disorders and migraine comorbidities, including major depressive disorder and bipolar disorder [10]. Evening chronotypes typically prefer later sleep and wake times, accumulate sleep debt during the workweek, and often experience misalignment between biological and occupational schedules, which may contribute to increased susceptibility to mood disturbances and impaired physical and cognitive functioning [4].

Given these considerations, the current study aimed to determine the distribution of chronotypes among patients diagnosed with TTH, and to examine the associations between chronotype characteristics and pain intensity, sleep quality, depression, and quality of life. Additionally, the study sought to explore whether headache attacks tend to peak at specific times of day (circadian pattern) in relation to chronotype and whether headache frequency differs according to chronotype.

## 2. Materials and Methods

### 2.1. Study Design and Setting

This cross-sectional observational study was conducted at SANKO University Sani Konukoğlu Hospital Health Practice and Research Centre. The study adhered to the ethical principles of the Declaration of Helsinki and received approval from the SANKO University Non-Invasive Research Ethics Committee (approval no: 04/2021/16). Reporting of the study followed the STROBE (Strengthening the Reporting of Observational Studies in Epidemiology) checklist.

### 2.2. Study Participants

Adult patients diagnosed with TTH according to the International Classification of Headache Disorders-III (ICHD-III) were consecutively recruited from the neurology outpatient clinic of the hospital between June 2021 and June 2022 [11]. A total of 77 patients meeting the inclusion criteria and providing consent were included in the study. Patients were excluded if they were under 18 years of age, were receiving medication for any psychiatric disorder, had a diagnosed sleep disorder (e.g., obstructive sleep apnea, restless leg syndrome), used analgesic medication regularly for any reason, had a history of medication-overuse headache, or had received antidepressant treatment within the preceding month. A post hoc power analysis was performed based on the observed PSQI group means and standard deviations (Evening chronotype: 9.63 ± 5.58, Intermediate chronotype: 7.98 ± 4.05, Morning chronotype: 5.14 ± 3.06). For the three groups with a total of 77 participants, the calculated effect size was Cohen’s f = 0.366, indicating that approximately 11.8% of the total variance was explained by group differences (η^2^ = 0.118), which corresponds to a medium-to-large effect. Given this effect size and an alpha level of 0.05, the statistical power (1 − β) was estimated to be approximately 0.82. Therefore, the current sample size provided sufficient power to detect differences in the observed magnitude [12,13].

### 2.3. Data Collection

Data were collected through in-person interviews using the following instruments:

Sociodemographic data form: This structured form collected information on demographic characteristics (age, sex, education level, height, body weight, body mass index (BMI), smoking and alcohol use), clinical features (headache duration, frequency, duration of attacks, precipitating factors, analgesic use and response, known comorbidities, and neurological findings), and work schedule. Headache frequency was recorded as ≤4 or >4 attacks per month. 

Visual Analogue Scale (VAS): Pain intensity was self-rated by the participants on a 0–10 scale, where 0 indicates no pain and 10 indicates the worst imaginable pain [14,15].

Morningness–Eveningness Questionnaire (MEQ): The MEQ is a self-reported questionnaire used to assess individual preferences for morningness and eveningness. Chronotype of the participants was classified based on total MEQ scores. Respondents indicate their preferred times for sleep, wake, and activity, reflecting their internal circadian rhythm rather than their actual schedule. The questionnaire consists of 19 items, each scored on a 4–5 point multiple-choice scale. Total scores range from 16 to 86. Scores ≤ 41 indicate an evening type, scores ≥ 59 indicate a morning chronotype, and scores between 42 and 58 indicate an intermediate chronotype [16]. The Turkish adaptation of the tool showed a test–retest reliability coefficient of 0.84 [17]. 

These items illustrate the preferential nature of the MEQ and the method used to classify participants as morning, intermediate, or evening chronotypes.

Hospital Anxiety and Depression Scale (HADS): The HADS is a 14-item self-report questionnaire developed by Zigmond and Snaith to assess anxiety and depressive symptoms (7 items each) [18]. Each item is scored from 0 to 3, yielding a total score of 0–21 for either anxiety or depression. Scores of 8–10 indicate a moderate level of symptoms, while scores above 11 suggest a clinically significant level of symptoms that may correspond to a clinical diagnosis [18]. Validity and reliability of the Turkish version of the scale were demonstrated by Aydemir et al., with Cronbach’s alpha coefficients of 0.85 for the anxiety subscale and 0.77 for the depression subscale [19].

Pittsburgh Sleep Quality Index (PSQI): The PSQI is a widely used self-report questionnaire that assesses sleep quality and disturbances over the previous month [20]. It consists of 19 items generating seven component scores: subjective sleep quality, sleep latency, sleep duration, habitual sleep efficiency, sleep disturbances, use of sleep medication, and daytime dysfunction. Each component is scored from 0 to 3. The component scores are summed to yield a total score ranging from 0 to 21. The original developers reported an internal consistency of Cronbach’s alpha = 0.83, a test–retest reliability of 0.85 for the global scale, a sensitivity of 89.6%, and a specificity of 86.5% [20]. The Turkish version developed by Ağargün et al. demonstrated good internal consistency (Cronbach’s alpha = 0.80). The total PSQI score clearly distinguishes between good sleepers (total score ≤ 5) and poor sleepers (total score > 5) [21]. The questionnaire has been validated in various clinical populations, including patients with major depressive disorder, sleep disorders, cancer, and fibromyalgia [22].

Short Form 36 (SF-36): The SF-36 is a self-assessment questionnaire developed to evaluate health-related quality of life [23]. It consists of 36 items across eight dimensions: physical functioning (10 items), social functioning (2 items), role limitations due to physical problems (4 items), role limitations due to emotional problems (3 items), mental health (5 items), energy/vitality (4 items), bodily pain (2 items), and general health perceptions (5 items) The assessment considers experiences over the past four weeks and is widely used to compare general and specific populations, evaluate the effects of interventions, and monitor patients over time.

Validity and reliability of the Turkish SF-36 questionnaire have been demonstrated by Kocyigit et al. [24]. The Turkish SF-36 demonstrated high internal consistency, with Cronbach’s alpha values for the subscales ranging from 0.792 to 0.992. Item-total correlations were: physical functioning 0.436–0.840, role limitations (physical) 0.887–0.895, bodily pain 0.861–0.958, general health perceptions 0.564–0.892, vitality 0.702–0.841, social functioning 0.949–0.952, role limitations (emotional) 0.396–0.473, and mental health 0.456–0.824. Convergent validity was supported by significant correlations between SF-36 subscales and the Turkish HADS, confirming the scale’s sensitivity to both physical and mental health dimensions [24].

### 2.4. Variables and Outcomes

Dependent Variables (Outcomes): The primary outcomes of interest were pain intensity, assessed using the VAS, and bodily pain, measured by the SF-36 Bodily Pain (BP) subscale. These outcomes were selected as they represent the core clinical manifestations of TTH and are widely used in headache and chronic pain research.

Independent Variables (Predictors): The primary explanatory variable was chronotype, assessed by the MEQ score. MEQ scores were treated both as a numeric variable and as categorical groups (evening, intermediate, and morning chronotypes) for descriptive and inferential analyses. Secondary predictors (covariates) included sex, HADS-A score (anxiety), and HADS-D score (depression).

Headache-Specific Variables: These variables included duration of pain (in months), duration of TTH attacks (hours), frequency of TTH attacks (number per month), circadian pattern of attacks, and analgesic use and response. Frequency of TTH attacks was further dichotomized as ≤4 or >4 per month based on prior literature indicating that ≥4 attacks per month represents a clinically relevant threshold for recurrent TTH [25]. These variables were collected for descriptive purposes and were not treated as explanatory variables in the regression models.

Descriptive Sociodemographic and Clinical Variables: Sociodemographic and clinical characteristics, including age, sex, BMI, education level, smoking status, alcohol use, work schedule (shift work), comorbidities, were collected to describe the sample.

Outcomes for Regression Models: Two separate linear regression models were constructed:

- Model 1: Outcome = SF-36 BP score
- Model 2: Outcome = VAS score

### 2.5. Statistical Methods

Continuous variables were reported as mean ± standard deviation (SD) when normally distributed and as median [25th–75th percentile] when non-normally distributed. Group differences in normally distributed continuous variables (e.g., BMI) were evaluated using one-way analysis of variance ANOVA with Tukey’s post hoc tests for pairwise comparisons. Non-normally distributed or ordinal variables (e.g., frequency of analgesic use and analgesic response) were analyzed using the Kruskal–Wallis test. Categorical variables, including the circadian pattern of headache attacks, were compared using contingency tables, with Fisher’s exact test applied when expected cell counts were low.

Primary analyses were conducted using linear regression models to examine associations between predictors and outcomes. Dependent variables (outcomes) included VAS and SF-36 BP scores. These outcomes were chosen as they represent the core clinical manifestations of TTH and are widely used in headache and chronic pain research. The primary explanatory variable was MEQ score (chronotype), treated as a numeric variable, while sex, HADS-A score, and HADS-D score were included as covariates to account for potential confounding effects. The models did not include MEQ score as a moderator or mediator. These variables were included in the regression models because SF-36 BP score represents the pain component of quality of life, VAS score reflects subjective pain intensity, and prior literature indicates that sex, anxiety, and depression are associated with pain perception and chronotype differences in headache and chronic pain populations [8,9]. Age was evaluated in preliminary analyses but was not included as a covariate because it did not show significant associations with the outcomes. Standardized beta coefficients (β) were reported to allow direct comparison of the relative magnitude of associations across predictors. Separate models were constructed for each outcome (Model 1: SF-36 BP; Model 2: VAS). Model performance was evaluated using R^2^ and Akaike Information Criterion (AIC), with lower AIC values indicating better fit while penalizing additional variables.

All statistical tests were two-tailed, and *p*-values < 0.05 were considered statistically significant. All analyses were conducted using R software version 3.6.0 (R Core Team, 2019; R Foundation for Statistical Computing, Vienna, Austria).

## 3. Results

### Demographics and Clinical Characteristics

A total of 77 participants, including 63 women and 14 men aged 18 to 52, were included in the study. Fifty-five participants (71.4%) had an intermediate chronotype, 14 (18.2%) had a morning chronotype, and 8 (10.4%) had an evening chronotype (Table 1).

No significant differences in age, sex, education level, smoking, alcohol use, shift work, comorbidities, pain duration, duration or frequency of TTH attacks were observed across chronotype groups.

A one-way ANOVA indicated a significant effect of chronotype on BMI (F(2, 74) = 3.52, *p* = 0.035, η^2^ = 0.087) (Table 1). Post-hoc comparisons showed that participants with an evening chronotype had a significantly lower BMI than those with an intermediate chronotype (*p* = 0.033) and a trend toward lower BMI relative to morning chronotypes (*p* = 0.053). No significant difference was observed between the morning and intermediate chronotypes.

A contingency table analysis of the circadian pattern of TTH attacks across chronotype groups showed that 37.5% (*n* = 3) of participants with an evening chronotype exhibited a circadian pattern, compared with 74.5% (*n* = 41) of those with an intermediate chronotype and 78.6% (*n* = 11) of those with a morning chronotype. Fisher’s exact test indicated that this association was not significant (*p* = 0.089) (Table 1). The timing of peak headache intensity varied by chronotype. Among evening chronotypes, peak intensity was evenly distributed across early morning (00:00–06:00, 33%), morning (06:00–12:00, 33%), and afternoon (12:00–18:00, 33%) intervals. For participants with a morning chronotype, peak intensity was most frequently reported in the evening (18:00–00:00, 45%), followed by the afternoon (12:00–18:00, 36%), with fewer reports in the early morning (9%) and morning (9%) intervals. In the intermediate chronotype group, peak intensity was most common in the afternoon (12:00–18:00, 34%) and evening (18:00–00:00, 29%), and less frequent in the morning (22%) and early morning (15%) intervals.

Analysis of analgesic use frequency across chronotype groups showed that among participants with an evening chronotype, most reported no use (62.5%), while rare use, use once every few weeks, or once a week were equally reported (12.5% each). In the intermediate chronotype group, the majority reported no use (56.4%), followed by rare use (18.2%), occasional use once every few weeks or once a week (1.8% each), use once every few days (9.1%), once daily (3.6%), and several times daily (9.1%). Among participants with a morning chronotype, most reported no use (71.4%), followed by rare use (14.3%), and occasional use once every few weeks or once every few days (7.1% each). Overall, the majority of participants (59.7%) reported no analgesic use. A Kruskal–Wallis test revealed no significant differences among the chronotype groups (Table 1).

The most commonly reported analgesic response was “occasional” (Evening chronotype: 75.0%; Intermediate chronotype: 58.2%; Morning chronotype: 50.0%). Kruskal–Wallis test showed no significant differences across chronotype groups (Table 1).

A Kruskal–Wallis test indicated a significant difference in SF-36 Role limitations due to physical problems (RP) scores across chronotype groups (ε^2^ = 0.0852). Post-hoc Dwass–Steel–Critchlow–Fligner comparisons showed that the evening chronotypes scored significantly lower than the morning chronotypes (W = 3.47, *p* = 0.038), with no significant differences between the evening and intermediate chronotypes (W = 2.37, *p* = 0.214) or intermediate and morning (W = 2.32, *p* = 0.229) chronotypes (Table 2).

Linear regression analysis using SF-36 BP score as the dependent variable, MEQ score (chronotype) as the primary predictor, and sex, HADS-A, and HADS-D scores as covariates yielded a significant model, with all model assumptions satisfactorily met (Model 1, Table 3).

A separate linear regression model using VAS score as the dependent variable and MEQ score, sex, HADS-A, and HADS-D scores as predictors was also significant. MEQ score (chronotype) was not a significant predictor, whereas sex was significant, with higher VAS scores observed in females (Model 2, Table 3).

Supplementary Table S1 shows the correlation matrix that illustrates the relationships between chronotype, anxiety, depression, pain and quality of life.

## 4. Discussion

This study investigated the associations between chronotype, depression, anxiety, sleep quality, pain intensity, and quality of life in individuals with TTH. A greater prevalence of negative outcomes was observed among individuals with an evening chronotype. Evening chronotypes typically exhibit delayed sleep and wake times and greater variability in sleep–wake patterns, which can predispose them to impaired daytime functioning and increased pain sensitivity [26]. The majority of patients in this study exhibited an intermediate chronotype, while fewer participants showed characteristics of morning or evening chronotypes. The relationship between chronotype and other primary headache types, such as migraine and cluster headache, has been investigated, yielding inconsistent findings. Although anecdotal evidence suggests that migraineurs are more likely to have an evening chronotype, few studies support this hypothesis [27]. In our previous study of a migraine cohort, contrary to earlier reports, the morning chronotype was significantly more common than the evening chronotype, suggesting that chronotype distribution in migraine can be heterogeneous [28]. Cluster headaches have been linked to circadian rhythm disturbances, though no direct association with chronotype has been demonstrated [29]. As few studies have examined the relationship between TTH and chronotype, our findings contribute to address this important gap in the literature.

Previous studies have indicated that individuals with an evening chronotype tend to have a significantly higher BMI and an increased risk of obesity, attributed to internal circadian rhythm misalignment, shorter sleep duration, and irregular eating habits [30,31]. In contrast, our study found that patients with an evening chronotype had a significantly lower BMI. This inconsistency may be explained by the relatively younger age of our sample and the predominance of female participants. Prior research also suggested that the relationship between chronotype and BMI may vary by age, sex, and population characteristics [32]. Furthermore, lifestyle factors such as meal timing, caffeine intake, and physical activity, which were not systematically evaluated in this study, could have influenced these findings. Future studies incorporating objective assessments of sleep and metabolic parameters are warranted to clarify these associations.

Analysis of the circadian pattern of TTH attacks and the timing of peak headache intensity revealed some differences across chronotypes. Participants with morning and intermediate chronotypes more frequently reported peak intensity in the afternoon and evening, whereas evening chronotypes exhibited a more evenly distributed pattern throughout the day. However, the very small number of evening chronotypes (*n* = 3) limits the reliability of these observations, and Fisher’s exact test indicated no statistically significant association (*p* = 0.089). Previous studies exploring the effects of chronotype on headache characteristics revealed that morning chronotype usually correlated with migraine attacks starting in the morning, whereas evening chronotype correlated with attacks in the afternoon/evening and with higher attack frequency [5,33]. In contrast, in patients with TTH, time preference of headache attacks and headache frequency were not significantly associated with chronotype, consistent with the findings of the present study [33]. While these trends do not reach statistical significance, they suggest that chronotype may influence the timing of headache peaks in a manner potentially relevant for individualized symptom monitoring or intervention planning. For example, morning chronotypes may be more prone to experiencing headache intensity later in the day, which could inform timing for pharmacologic or behavioral strategies aimed at headache management.

Social jetlag, defined as the difference in sleep midpoint between workdays and free days, arises when an individual’s biological rhythm is misaligned with the socially imposed schedule of activity and rest. This misalignment represents a chronic stressor linked to increased risk of physical and mental health disorders [4,34,35]. In our study, a significant negative correlation was found between chronotype and sleep quality (PSQI), indicating poorer sleep quality with increasing evening preference, consistent with existing literature [36]. Sleep deprivation and susceptibility to sleep deprivation can precipitate headaches [37]. The reason for inadequate sleep in TTH is reportedly a higher level of insomnia compared to healthy individuals [38]. A significant association was also demonstrated by İpar et al. between poor sleep quality, evening chronotype, and an increased likelihood of depression among Turkish youth aged 18 to 24 years [39]. Similarly, a more recent study showed that evening-type individuals experience delayed sleep onset and poorer subjective sleep quality [26].

In the current study, evening chronotypes scored significantly lower on the SF-36 “Role Limitations Due to Physical Problems” subdomain (RP), indicating greater limitations in daily activities. In contrast, scores in the Physical Functioning (PF) domain did not differ significantly across chronotypes, suggesting that general physical capacity was largely preserved. These results imply that evening chronotypes may face role-specific limitations rather than broad reductions in physical ability, aligning with previous research reporting higher disability rates and lower quality of life among evening chronotypes [6,40].

Although no statistically significant differences were found between chronotypes in other SF-36 subdomains, such as social functioning or mental health, evening-type individuals consistently reported lower scores. These findings align with previous reports linking evening chronotype to reduced well-being, excessive daytime sleepiness, and insufficient sleep duration [41].

Regarding the results of regression analyses, while no significant differences were observed between chronotypes in depression (HADS-D) or pain intensity (VAS), sex showed a significant association with pain outcomes. Consistently, studies have shown significantly higher VAS scores in women with migraine or TTH. This finding was attributed to hormonal influences and more pessimistic coping strategies among women [42,43]. Linear regression analysis also revealed that chronotype (MEQ score) significantly predicted SF-36 Bodily Pain scores, independent of anxiety and depression. In contrast, chronotype did not significantly predict VAS pain intensity. These findings suggest that chronotype may be associated with the perceived impact of pain on daily functioning rather than direct pain intensity.

Overall, this study provides novel insights into the multifaceted effects of chronotype in individuals with TTH. Limitations include the cross-sectional design, small overall sample, and very limited representation of evening chronotypes. Despite these limitations, the study contributes meaningful evidence to an underexplored area and underscores the potential relevance of chronobiological characteristics in the management of TTH.

## 5. Conclusions

Evening chronotype was associated with poorer sleep quality and greater role limitations due to physical problems. Chronotype did not directly predict pain intensity, whereas sex was a significant predictor of pain intensity. These findings underscore the relevance of chronobiological characteristics and sleep–pain interactions in individuals with TTH. Interventions aimed at improving sleep quality and aligning circadian rhythms may have the potential to enhance patient-reported outcomes. Future longitudinal studies with larger samples, objective assessments of sleep and circadian rhythms, and consideration of hormonal and lifestyle factors are warranted to further clarify these relationships and inform individualized approaches to TTH management.

## Figures and Tables

**Table 1:** Demographic and Clinical Characteristics of the Participants.

Variable	Evening Chronotype (*n* = 8)	Intermediate Chronotype (*n* = 55)	Morning Chronotype (*n* = 14)	Total (*n* = 77)	Test	*p*-Value
Age, years	23.5 [20.8–27.8]	28 [24–33]	34.5 [32.3–38.5]	29.0 [24.0–35.0]	6.92 *	0.031
Sex, female	5 (62.5%)	39 (70.9%)	19 (71.4%)	54 (70.1%)	^†^	0.921
BMI, kg/m^2^	21.7 (2.88)	25.2 (4.5)	26.5 (4.22)	25.2 (4.47)	3.52 ^‡^	0.035
Education level, University degree	2 (25%)	18 (32.7%)	6 (42.9%)	26 (33.8%)	0.02 *	0.987
Smoking, yes	4 (50%)	24 (43.6%)	5 (35.7%)	33 (42.9%)	^†^	0.814
Alcohol use, yes	1 (12.5%)	5 (9.1%)	0	6 (7.8%)	^†^	0.380
Work schedule, shift work	1 (12.5%)	7 (12.7%)	1 (7.1%)	8 (11.7%)	^†^	0.960
Comorbidities, yes	0	9 (16.4%)	3 (21.4%)	12 (15.6%)	^†^	0.464
Pain duration, <1 year	3 (37.5%)	25 (45.5%)	6 (42.9%)	34 (44.2%)	^†^	0.345
Duration of TTH attacks, 4–24 h	6 (75.0%)	30 (54.5%)	8 (57.1%)	44 (57.1%)	^†^	0.790
Frequency of TTH attacks, >4 per month	7 (87.5%)	40 (72.7%)	9 (64.3%)	56 (72.7%)	^†^	0.549
Circadian pattern of TTH attacks, yes	3 (37.5%)	41 (74.5%)	11 (78.6%)	55 (71.4%)	^†^	0.089
Frequency of analgesic use, No use	5 (62.5%)	31 (56.4%)	10 (71.4%)	46 (59.7%)	1.57 *	0.457
Analgesic response, Occasional	6 (75%)	32 (58.2%)	7 (50%)	45 (58.4%)	1.01 *	0.603

Note. Data are presented as mean (± standard deviation) or median [25th percentile–75th percentile] for continuous variables and number (percentage) for categorical variables. * Kruskal–Wallis test (χ^2^ value), ^†^ Fisher’s exact test, ^‡^ ANOVA F-value. Abbreviations. BMI: Body mass index.

**Table 2:** Assessment Scale Scores by Chronotype.

Variable	Evening Chronotype (*n* = 8)	Intermediate Chronotype (*n* = 55)	Morning Chronotype (*n* = 14)	Test	*p*-Value
HADS-A	12.88 (5.69)	11.93 (4.76)	9.07 (5.53)	1.72 *	0.213
HADS-D	10.25 (3.73)	8.80 (4.19)	7.79 (4.96)	0.853 *	0.445
VAS	7 [5–9]	7 [5–9]	6 [4–8]	2.08 ^†^	0.354
PSQI-Global	9.63 (5.58)	7.98 (4.05)	5.14 (3.06)	4.74 *	0.024
SF-36, PF	72.5 [58.8–88.8]	80 [65–95]	77.5 [57.5–97.5]	0.321 ^†^	0.852
SF-36, RP	25 [0–25]	50 [25–75]	75 [50–100]	6.48 ^†^	0.039
SF-36, RE	33.3 [0–33.3]	33.3 [0–100]	100 [41.7–100]	4.74 ^†^	0.093
SF-36, VT	26.3 (14.6)	38.8 (21.2)	42.9 (21.9)	2.66 *	0.097
SF-36, MH	43.0 (22.0)	47.3 (21.4)	53.1 (24.7)	0.508 *	0.612
SF-36, SF	50 [34.4–50]	50 [37.5–75]	56.3 [40.6–62.5]	3.15 ^†^	0.207
SF-36, BP	39.7 (20.7)	42.9 (23.0)	52.3 (19.7)	1.39 *	0.277
SF-36, GH	49.4 (21.5)	47.1 (18.0)	51.8 (17.7)	0.387 *	0.686

Note. Data are presented as mean (± standard deviation) or median [25th percentile–75th percentile]. * ANOVA (Welch’s) F-value, ^†^ Kruskal–Wallis (χ^2^ value). Abbreviations. HADS-A: Hospital Anxiety Depression Scale-Anxiety, HADS-D: Hospital Anxiety Depression Scale-Depression, VAS: Visual Analogue Scale, PSQI: Pittsburgh Sleep Quality Index, SF-36: Short Form-36, PF: Physical Functioning, RP: Role Limitations due to Physical Problems, RE: Role Limitations due to Emotional Problems, VT: Energy/Vitality, MH: Mental Health, SF: Social Functioning, BP: Bodily Pain, GH: General Health Perceptions.

**Table 3:** Linear Regression Models.

Variable	β (Standardized Regression Coefficient)	95%CI	t	*p*-Value
Model 1 (Outcome = SF36_BP_)				
MEQ score	0.252	0.045 to 0.459	2.431	0.018
HADS-A score	−0.204	−0.478 to 0.070	−1.484	0.142
HADS-D score	−0.106	−0.366 to 0.154	−0.814	0.418
Sex	−0.630	−1.081 to −0.178	−2.781	0.007
Model statistics	F(4,72) = 7.90, *p* < 0.001; R^2^ = 0.305; AIC = 680			
Model 2 (Outcome = VAS)				
MEQ score	−0.192	−0.404 to 0.021	−1.800	0.076
HADS-A score	0.206	−0.076 to 0.487	1.458	0.149
HADS-D score	0.010	−0.257 to 0.277	0.075	0.940
Sex	0.773	0.309 to 1.236	3.322	0.001
Model statistics	F(4,72) = 6.54, *p* < 0.001; R^2^ = 0.266			

Abbreviations: CI: Confidence Interval, HADS-A: Hospital Anxiety Depression Scale-Anxiety, HADS-D: Hospital Anxiety Depression Scale-Depression, VAS: Visual Analogue Scale, SF-36: Short Form-36, BP: Bodily Pain.

## Data Availability

Due to restrictions (data protection, legal or ethical reasons), the data is available on request.

## Supplementary Materials

The following supporting information can be downloaded at: https://www.mdpi.com/article/10.3390/healthcare13222902/s1, Table S1: Correlation Matrix.
