# Supplementary material for: Associations Between Chronotype and Pain, Sleep Quality, Depression, and Quality of Life in Patients with Tension-Type Headache
Source: Healthcare (Basel). 2025 Nov 14;13(22):2902. doi: 10.3390/healthcare13222902 (PMC12652725; doi:10.3390/healthcare13222902)
Supplement: Supplementary file 1 [file healthcare-13-02902-s001.zip › healthcare-3892045-supplementary.pdf]

Supplementary Material

Table S1. Correlation Matrix

		MEQ	Age	BMI	HADS-A	HADS-D	SF-36, PF	SF-36, RP	SF-36, RE	SF-36, VT	SF-36, MH	SF-36, SF	SF-36, BP	SF-36, GH	VAS
**MEQ**	***r***	-													
	p	-													
**Age**	***r***	0.382	-												
	p	**<0.001**	-												
**BMI**	***r***	0.284	0.483	-											
	p	**0.012**	**<0.001**	-											
**HADS-A**	***r***	-0.276	-0.088	-0.090	-										
	p	**0.015**	0.447	0.436	-										
**HADS-D**	***r***	-0.204	0.068	-0.067	0.657	-									
	p	0.076	0.558	0.564	**<0.001**	-									
**SF-36, PF**	***r***	0.084	-0.162	0.056	-0.284	-0.277	-								
	p	0.468	0.158	0.630	**0.012**	**0.015**	-								
**SF-36, RP**	***r***	0.281	0.060	0.168	-0.357	-0.503	0.236	-							
	p	**0.013**	0.602	0.145	**0.001**	**<0.001**	**0.039**	-							
**SF-36, RE**	***r***	0.211	0.090	0.201	-0.383	-0.483	0.210	0.737	-						
	p	0.066	0.438	0.080	**<0.001**	**<0.001**	0.067	**<0.001**	-						
**SF-36, VT**	***r***	0.260	0.017	0.157	-0.540	-0.643	0.282	0.269	0.334	-					
	p	**0.022**	0.881	0.172	**<0.001**	**<0.001**	**0.013**	**0.018**	**0.003**	-					
**SF-36, MH**	***r***	0.199	0.121	0.175	-0.610	-0.607	0.334	0.323	0.343	0.754	-				
	p	0.082	0.293	0.128	**<0.001**	**<0.001**	**0.003**	**0.004**	**0.002**	**<0.001**	-				
**SF-36, SF**	***r***	0.188	0.081	0.155	-0.427	-0.416	0.145	0.145	0.505	0.399	0.420	-			
	p	0.101	0.483	0.179	**<0.001**	**<0.001**	0.208	0.208	**<0.001**	**<0.001**	**<0.001**	-			
**SF-36, BP**	***r***	0.310	0.069	0.078	-0.429	-0.349	0.290	0.446	0.346	0.436	0.439	0.597	-		
	p	**0.006**	0.552	0.497	**<0.001**	**0.002**	**0.011**	**<0.001**	**0.002**	**<0.001**	**<0.001**	**<0.001**	-		
**SF-36, GH**	***r***	0.147	0.051	0.186	-0.402	-0.464	0.478	0.289	0.335	0.522	0.480	0.280	0.344	-	
	p	0.201	0.659	0.106	**<0.001**	**<0.001**	**<0.001**	**0.011**	**0.003**	**<0.001**	**<0.001**	**0.014**	**0.002**	-	
**VAS**	***r***	-0.226	-0.149	-0.146	0.371	0.254	-0.288	-0.277	-0.365	-0.303	-0.374	-0.345	-0.324	-0.247	-
	p	**0.048**	0.195	0.206	**<0.001**	**0.026**	**0.011**	**0.015**	**0.001**	**0.007**	**<0.001**	**0.002**	**0.004**	**0.030**	-

Note. Statistically significant p values are denoted in bold.

Abbreviations. *r:* Pearson's r value, BMI: Body mass index, HADS-A: Hospital Anxiety Depression Scale - Anxiety, HADS-D: Hospital Anxiety Depression Scale - Depression, SF-36: Short Form-36, PF: Physical Functioning, RP: Role Limitations due to Physical Problems, RE: Role Limitations due to Emotional Problems, VT: Energy/Vitality, MH: Mental Health, SF: Social Functioning, BP: Bodily Pain, GH: General Health Perceptions, VAS: Visual Analogue Scale.
